# The Fat-Pancreas Axis: A Narrative Review Unraveling the Role of Obesity in Pancreatic Diseases

**DOI:** 10.7759/cureus.99148

**Published:** 2025-12-13

**Authors:** George S Zacharia, FNU Veena, Huzefa Habib, Allah Dad, Michael Asare

**Affiliations:** 1 Internal Medicine, BronxCare Health System, New York, USA; 2 Gastroenterology and Hepatology, Ahalia Hospital Mussafah, Abu Dhabi, ARE; 3 Internal Medicine, King Edward Medical University, Lahore, PAK; 4 Internal Medicine, Sheikh Zayed Medical College, Rahim Yar Khan, PAK

**Keywords:** bmi, obesity, overweight, pancreatic cancer, pancreatitis

## Abstract

Obesity has alarmingly risen across the globe as a significant public health concern and is increasingly recognized as a complex systemic disease with widespread metabolic and inflammatory consequences. While its associations with cardiovascular, hepatic, and metabolic disorders are well established, the impact of obesity on the pancreas has received comparatively less attention. Emerging evidence highlights that pancreatic steatosis is closely linked to obesity, type 2 diabetes, metabolic syndrome, and fatty liver disease. Fatty pancreas contributes to both endocrine and exocrine dysfunction through lipotoxicity, oxidative stress, and adipokine-mediated inflammation. In acute pancreatitis, obesity exacerbates disease severity by amplifying systemic inflammatory responses, promoting lipolysis of peripancreatic fat, and delaying immune resolution, all of which increase complications and mortality. The role of obesity in chronic pancreatitis is less defined, though intrapancreatic fat accumulation appears to predispose to parenchymal injury and progression. Obesity is increasingly considered an independent risk factor for pancreatic cancer, with excess adiposity driving neoplastic transformation through chronic inflammation, insulin resistance, hyperinsulinemia, and activation of oncogenic pathways. Weight reduction is associated with reduced pancreatic steatosis and may lower risk; however, causal data are limited, and further longitudinal studies are required. Taken together, obesity represents a major modifiable determinant of pancreatic health, with implications spanning steatosis, inflammatory diseases, and malignancy. Increasing scientific data on the obesity-pancreatic diseases conundrum also necessitates the need for integrating obesity-related markers into risk stratification and prognostic models, as well as exploring targeted therapeutic strategies in obesity-associated pancreatic disease.

## Introduction and background

The World Health Organization (WHO) defines obesity as a body mass index (BMI) of 30 kg/m² or higher, while those with a BMI ranging from 25 kg/m² to 30 kg/m² are classified as overweight. In 1997, given the alarming rise in incidence and prevalence, the WHO declared obesity an epidemic [1]. According to the 2023 World Obesity Atlas report, 38% of the global population is currently overweight or obese [2]. It is predicted that by 2030, 78% of the United States (US) adult population and by 2035, half of adults globally will be obese or overweight [2,3]. Obesity has far outgrown from a mere cosmetic or nutritional concern to a complex systemic disease with extensive health ramifications. The list of pathologies or diseases linked to obesity is ever growing, including, but not limited to, type 2 diabetes mellitus (T2DM), hyperlipidemia, coronary, peripheral, and cerebrovascular diseases, osteoarthritis, steatotic liver diseases, sleep apnea and hypoventilation syndromes, and most neoplasia; all of these together contribute to nearly three million deaths a year across the globe [4]. The obesity-associated tissue lipid infiltration, adipokine production, lipotoxicity, and activation of the inflammatory cascade are believed to drive these sequelae [5,6]. Obesity-related insulin resistance and hyperinsulinemia also contribute to the deleterious effects [7].

Objectives

The impact of obesity on the pancreas has garnered less attention; however, of late, the small intra-abdominal organ is increasingly recognized as a target of obesity-related injury. Multiple studies claim an association between obesity and pancreatic diseases, including fatty pancreas, acute pancreatitis, and pancreatic adenocarcinoma; however, most are yet to be substantiated unequivocally. The narrative review aims to (a) synthesize current evidence on the impact of obesity on the spectrum of pancreatic diseases, (b) briefly analyze the pathophysiological mechanisms linking obesity to pancreatic injury, and (c) familiarize the concept of fatty pancreas or pancreatic steatosis, its diagnosis, and implications.

Search strategy

We identified potential references for this narrative review using a search of Google Scholar and PubMed for articles published until August, 2025 with the following search string(s): ("Obesity"[MeSH Terms] OR obesity [Title/Abstract] OR "overweight"[MeSH Terms] OR overweight [Title/Abstract] OR "body mass index"[Title/Abstract] OR BMI[Title/Abstract]) AND ("fatty pancreas"[Title/Abstract] OR "pancreatic steatosis"[Title/Abstract] OR "nonalcoholic fatty pancreas"[Title/Abstract] OR "non-alcoholic fatty pancreas"[Title/Abstract] OR "pancreatic fat"[Title/Abstract] AND ("pancreatic diseases"[MeSH Terms] OR "pancreatic disease"[Title/Abstract] OR "acute pancreatitis"[MeSH Terms] OR "acute pancreatitis"[Title/Abstract] OR "chronic pancreatitis"[MeSH Terms] OR "chronic pancreatitis"[Title/Abstract] OR "pancreatic neoplasms"[MeSH Terms] OR "pancreatic neoplasm"[Title/Abstract] OR "pancreatic cancer"[Title/Abstract] OR "pancreatic ductal adenocarcinoma"[Title/Abstract]).

Manuscripts in English reporting trials, reviews, and consensus on the association between obesity and pancreatic diseases were identified and reviewed by the authors. Given the objective of this manuscript, to provide a broad narrative overview rather than a quantitative synthesis, and due to substantial heterogeneity in study designs, populations, and outcome measures across the available literature, a systematic review was not undertaken. The derived information was organized into subsections: fatty pancreas, acute pancreatitis, chronic pancreatitis, and pancreatic neoplasia, with emphasis on the role of obesity in each condition.

## Review

Fatty pancreas

**Figure 1:**
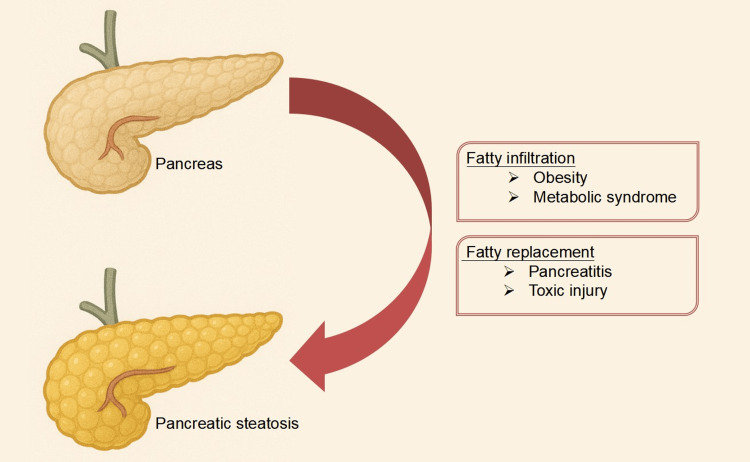
Fatty pancreas. Pancreatic steatosis results from either fatty infiltration or fatty replacement of normal pancreatic parenchyma. Fatty replacement is an essentially irreversible process secondary to pancreatic tissue loss; while fatty infiltration is a reversible process associated with obesity, metabolic syndrome, etc.

Fatty pancreas (FP), also known as pancreatic steatosis, is characterized by adipose infiltration of pancreatic tissue and is increasingly recognized with obesity, T2DM, metabolic syndrome, and fatty liver disease [8]. Pancreatic steatosis was first described by Ogilvie in 1933, when he observed a doubling of the amount of fat in the pancreas of obese individuals compared to their non-obese counterparts [9]. Non-alcoholic fatty pancreatic disease (NAFPD) is defined as pancreatic fat accumulation in the absence of significant alcohol intake: <20 gm/day ethanol use [10]. The estimated prevalence of fatty pancreatic diseases ranges between 11% and 33% among Asians [11,12]. A recent meta-analysis of 18 studies reported an overall prevalence of 21.1% (CI: 11.04%-36.58%); however, prevalence varied across diagnostic tests [13]. Beyond fat infiltration, fatty replacement is an alternate mechanism of fat in the pancreas; here, the dead or injured pancreatic acinar cells get replaced by adipose tissue (Figure 1) [14].

Non-alcoholic fatty liver disease is the most critical predictor of NAFPD, suggesting that the diseases potentially share common risk factors. Obesity is considered the single most important risk factor for FP [15]. Metabolically active pancreatic fat and free fatty acids induce adipocyte-derived inflammatory factors, including tumor necrosis factor-α (TNF-α), interleukin-6 (IL-6), and monocyte chemoattractant protein-1 (MCP-1). The fatty infiltration and subsequent inflammatory milieu culminate in acinar loss injury/loss, apoptosis, inflammatory infiltration, and a decrease in islet β/α-cell ratio, leading to pancreatic exocrine and endocrine dysfunction [8].

The gold standard for diagnosing FP is pancreatic biopsy [12]. Unlike in fatty liver, where fat accumulates within hepatocytes, in FP, the fat deposition is mainly extracellular in the pancreatic interstitium. Histological assessment is limited to autopsy or surgical resection specimens and biopsy samples [16]. Given the invasive nature of biopsy and risks of serious complications, non-invasive methods are often preferred for the diagnosis of FP.

Transabdominal ultrasound scans are the most widely accessible and cost-effective modality; a hyperechoic pancreas compared with the liver and kidneys is suggestive of the diagnosis. Sun et al. evaluated the diagnostic accuracy of transabdominal ultrasound using both a conventional visual method and a deep learning method in patients with histologically proven pancreatic steatosis. The diagnostic accuracy of conventional visual ultrasound was average: 0.598 with an area under the curve (AUC) of 0.616 and a confidence interval (CI) of 0.512-0.713 in the training group, while the validation group had an accuracy of 0.738 with an AUC of 0.737 and a CI of 0.578-0.861. Adopting deep learning technology with ultrasound imaging, the accuracy improved to 0.814 (AUC: 0.901; CI: 0.839-0.962) and 0.857 (AUC: 0.837; CI: 0.708-0.966), respectively. The deep learning model also diagnosed pancreatic steatosis with a sensitivity and specificity of 92% and 76.5%, in the validation group [17]. The results of this study highlight the role of transabdominal ultrasound in FP and the advantages of artificial intelligence in diagnostic imaging. However, the diagnostic accuracy of conventional ultrasound results tends to be operator-dependent and may be limited by body habitus and bowel gas [8].

Endoscopic ultrasound (EUS) can overcome these limitations and allow pancreatic sampling for confirmation; however, it is not recommended solely for diagnosing FP due to its invasive nature. Sepe et al. graded the pancreas based on echogenicity in comparison with splenic parenchyma, ductal delineation, and the salt-and-pepper appearance of the pancreas on EUS. Pancreatic parenchyma, which is hypo/isoechoic (grade 1) or hyperechoic (grade 2) to splenic parenchyma, with clearly delineated ducts and clear salt-and-pepper dots, is regarded as normal pancreas. Moderate (grade 3) to severe (grade 4) hyperechoic parenchyma with moderate to severe loss of ductal delineation and salt-and-pepper dots are classified as FP [18]. Tanawat et al. proposed an EUS diagnostic criterion and compared it with histology. The criteria included hyperechoic parenchyma, fibrotic changes, and obscured salt-and-pepper appearance. Each parameter was scored according to a predefined protocol. The scoring system had a sensitivity of 96.2% (CI: 80.4-99.9) and specificity of 82.9% (CI: 72.0-90.8) in diagnosing FP [19].

Computed tomography (CT) also allows the diagnosis of FP. Non-contrast imaging is preferred, as intravenous contrast enhances the pancreas. Compared with a normal pancreas, FP appears hypodense; a cut-off of 36 Hounsfield units (HU) has been suggested by Kim et al. [15]. Previtali et al. evaluated quantitative CT imaging for diagnosing FP [20]. In pre-contrast images, the pancreatic attenuation was significantly lower in patients with FP (27 ± 20 HU) versus normal pancreas (38 ± 12 HU; p = 0.011), expressed as mean ± standard deviation (SD). A cut-off of 37.5 HU on non-contrast images yielded a sensitivity and specificity of 76% (CI: 59%-89%) and 66% (CI: 51%-82%) for the diagnosis of FP. During the portal venous phase of contrast CT, pancreatic attenuation < 83.5 HU had a sensitivity of 71% (CI: 51%-87%) and a specificity of 78% (CI: 60%-91%) for detecting fatty infiltration. FP is associated with increased pancreatic surface lobularity (PSL). The quantitative mean PSL in patients with fatty pancreas was 8.5 ± 1.6, compared with 6.6 ± 1.4 in patients with normal pancreas, expressed as mean ± SD (p = 0.001). A PSL threshold of 7.6 had a sensitivity of 75% (CI: 55%-89%) and a specificity of 81% (CI: 64%-93%) for identifying fatty infiltration of the pancreas. A combination of pancreatic attenuation of <83.5 HU on the portal venous phase of contrast abdominal CT with a quantitative PSL of >7.6 had an AUC of 0.83 (CI: 0.67-0.99) for the diagnosis of FP [20]. Estimation of the pancreatic index (PI) on quantitative CT (calculated as the pancreas-to-spleen CT density ratio) also helps diagnose pancreatic steatosis. A PI of ≤ 0.70 has a sensitivity and specificity of 79% in the diagnosis of FP [21].

Magnetic resonance imaging (MRI) offers additional advantages, including greater sensitivity and reduced radiation exposure. Proton magnetic resonance spectroscopy (MRS) is believed to have diagnostic accuracy similar to that of pancreatic histology [15]. The MRS technique provides the amount of fat in an abdominal organ, e.g., the pancreas, as a percentage in the range 0%-100%. Wu et al. estimated the average pancreatic fat content in healthy volunteers as 3.33% [13]. The pancreatic fat content estimated by the Dixon method correlates well with histological intra-parenchymal fat estimation (r = 0.81; p < 0.001) [22]. Livingstone et al. compared the Dixon method with MRS in healthy subjects and reported a strong correlation between the methods for estimating pancreatic fat fraction (r = 0.80, p < 0.0001) [23]. Yet another diagnostic tool, MRI proton density fat fraction (MRPDFF), enables the accurate quantification of pancreatic fat; a cut-off of 10.4% is considered diagnostic [24]. Schawkat et al. estimated the correlation between histological pancreatic lipomatosis and MRPDFF in patients who underwent pancreatoduodenectomy for adenocarcinoma. The study reported correlations of 0.668 (p < 0.001) and 0.707 (p < 0.001) at the resection margin and the pancreatic tail, respectively [25]. A pooled data analysis from nine studies, including 1209 healthy individuals, using MRI modalities, estimated the upper limit of pancreatic fat percentage at 4.48% ± 0.87%; the upper normal limit of pancreatic fat in healthy subjects was estimated to be 6.2% (two standard deviations above the mean) [12].

The consequences of FP involve both the endocrine and exocrine pancreas. Beta-cell dysfunction and apoptosis lead to diabetes mellitus (DM). Ethnic diversities, though unexplained, have been demonstrated in the degree of beta cell dysfunction in FP: higher impact in Hispanics, compared to African Americans and Caucasians [26]. The effect of fatty infiltration on exocrine function is less evaluated. Tahtacı et al. demonstrated lower fecal pancreatic elastase levels, a marker of exocrine pancreatic function, in patients with FP [27]. Lipotoxicity, oxidative stress, paracrine effects, and adipose tissue incited inflammatory response are the frequently postulated mechanisms leading to pancreatic dysfunction in patients with FP [8]. Multiple studies have reported an association between FP and pancreatic adenocarcinoma. A retrospective cohort study by Fukuda et al., including 183 patients, reported an association between FP, diagnosed using CT quantification of the pancreatic index, and pancreatic ductal adenocarcinoma. In this study, a pancreatic index of ≤0.7 was identified as an independent risk factor for pancreatic cancer (odds ratio: 2.31; p = 0.023) [21]. A large-scale prospective cohort study by Yamazaki et al., including nearly 30,000 subjects, using MRI to quantify pancreatic fat, reported a significant association between intrapancreatic fat deposition of >10% and pancreatic ductal adenocarcinoma (hazard ratio 3.35; p = 0.001), suggesting a causal link [26].

There is no standardized treatment for FP. Weight loss is associated with reduced pancreatic fat content [28,29]. Post-hoc analysis of data from the HELENA trial reported a statistically significant reduction in pancreatic fat content with calorie restriction at 12 weeks and 50 weeks [29]. A Finnish study reported a decrease in intrapancreatic fat after two weeks of exercise training in healthy individuals and in pre-DM/DM patients. The study used either sprint interval training or moderate-intensity continuous training as the exercise strategy [30]. Another study reported a statistically significant (p = 0.001) reduction in pancreatic fat content with a six-month moderate-intensity aerobic training program (−1.28 ± 2.34) compared to the control group (0.84 ± 3.0). The exercise group also showed improvement in β-cell function, as assessed by Homeostatic Model Assessment 2 for Insulin Resistance (HOMA2-IR), but the difference was not statistically significant [31].

Published literature suggests an improvement in NAFPD with metformin and troglitazone; however, the data are sparse [8,32,33]. The glucagon-like peptide-1 (GLP-1) receptor agonist liraglutide has been shown to significantly reduce pancreatic fat accumulation in patients with T2DM [34]. A combination of sitagliptin and telmisartan has been reported to improve pancreatic steatosis in mouse studies [35]. Chinese herbal medication containing berberine and cinnamic acid has demonstrated benefits in in vitro studies [36]. Bariatric surgeries are associated with improvement in pancreatic steatosis [8,37,38]. A meta-analysis by Wang et al. reported a mean reduction in intrapancreatic fat deposits by an absolute value of 3.9% (p = 0.003) following metabolic surgeries [39].

Acute pancreatitis

Acute pancreatitis (AP) is an acute inflammatory disease of the pancreas, typically presenting with abdominal pain radiating to the back, elevated pancreatic enzyme levels, and radiological evidence. The clinical spectrum ranges from mild, self-limiting inflammation to a life-threatening necrotizing disease with systemic inflammatory response syndrome (SIRS) and multiorgan failure [39]. Gallstones followed by alcohol and hypertriglyceridemia are the most common etiologies. Growing evidence suggests that obesity is associated with an increased risk of AP, exaggerated inflammatory response, and higher rates of local as well as systemic complications [40].

Obesity is associated with an increased risk of cholelithiasis and hypertriglyceridemia, the leading causes of AP. The increased cholesterol content in bile and lower circulating bile acid levels predispose to the development of biliary sludge and cholesterol stones [41]. Insulin resistance is the driving force behind hypertriglyceridemia in obese individuals [42]. The medications used in the setting of metabolic syndrome, obesity, and DM also predispose them to AP. Drug-induced pancreatitis has been reported with the use of GLP-1 agonists and dipeptidyl peptidase-4 (DPP-4) inhibitors [43,44].

The past decade witnessed an increasing role for GLP-1 agonists in the management of metabolic disorders, including DM, obesity, and metabolic-dysfunction-associated steatohepatitis (MASH), as well as cardio-renal protective effects. Multiple post-marketing case reports of AP have led to increasing concerns about the pancreatic safety of GLP-1 agonists. A recent meta-analysis reported a slightly increased risk of AP (relative risk: 1.44, 95% CI: 1.09-1.89; p = 0.009) with GLP-1 receptor agonists; however, this increased risk was not demonstrable when stratified by background medication use [45]. Ayoub et al. demonstrated that, when cohorts are matched for baseline demographics, comorbidities, and other medication use, there is no significant increase in risk of AP with GLP-1 agonist use [46]. Concerns about pancreatitis led to the avoidance of GLP-1 agonists in patients with a prior history of pancreatitis. Lomeli et al. evaluated the development of recurrent AP in patients with a past history of AP following initiation of GLP-1 agonists. Only 9.9% of patients experienced AP recurrence following GLP-1 exposure over 10 years, of which only 3.7% was attributable to GLP-1 agents [47]. The current FDA labels of GLP-1 agonists warn of pancreatitis as an adverse event and advise holding these agents promptly if AP develops and not restarting them in such cases; however, a prior history of pancreatitis is not regarded as a contraindication to starting GLP-1 agonists [48,49].

The bariatric interventions can also predispose to AP. Gastric bypass is associated with a 0.2% to 1% risk of AP, compared with 0.02% to 0.04% in the general population [50]. Cases of AP following placement of a gastric balloon have also been reported [51]. In addition to procedure-related acute or long-term mechanical complications, abrupt weight loss and dietary alterations, following weight-loss interventions lead to increased cholesterol content in bile, known as cholesterol supersaturation and biliary stasis, both of which contribute to biliary lithogenicity [52]. The cholesterol supersaturation predisposes to the development of biliary sludge and calculi, a risk factor for AP.

Obesity exacerbates AP through interrelated mechanisms. Excess adipose tissue contributes to elevated levels of pro-inflammatory cytokines, such as TNF-α, IL-6, and MCP-1, which collectively amplify SIRS and complicate AP [53]. The excess peripancreatic fat in visceral obesity undergoes lipolysis upon exposure to pancreatic enzymes, releasing toxic, unsaturated fatty acids. These free fatty acids induce lipotoxicity, resulting in oxidative stress, mitochondrial and endoplasmic reticulum dysfunction, inflammation, and further promote pancreatic acinar and endothelial cell death, thereby aggravating pancreatic necrosis. Studies in obese mouse models have shown that inhibition of lipolysis, during experimentally induced pancreatitis, prevents the rise in circulating unsaturated fatty acids and reduces renal and lung injury, systemic inflammation, hypocalcemia, pancreatic necrosis, and mortality [54]. In addition to perpetuating inflammation, obesity delays its resolution by disrupting immune regulation, thereby prolonging disease duration, severity, and complications in preclinical models. Emerging evidence indicates that obesity is a persistent immune dysregulator [55,56]. The prolonged immune response can contribute to the development of chronic pancreatitis. Animal studies also suggest that obesity may exacerbate inflammation by disrupting the intestinal mucosal barrier and altering the gut microbiota [57].

Beyond preclinical data, clinical studies also associate obesity, particularly visceral adiposity, with the severity and outcomes of AP [58,59]. In comparison to their normal-weight counterparts, those with elevated BMI have increased rates of local, systemic, and metabolic complications as well as an increased incidence of severe pancreatitis [58,60,61]. Funnell et al. prospectively compared and evaluated obese individuals (BMI >30 kg/m²) and non-obese individuals (BMI <30 kg/m²); the study demonstrated a higher risk of severe disease, pancreatic abscess formation, and mortality in obese individuals. The study reported that a BMI>30 kg/m² had a sensitivity of 63% and a specificity of 95% for predicting severe pancreatitis. Upon exclusion of biliary pancreatitis, the sensitivity to predict severe pancreatitis further increased to 86%, suggesting a better prognostic role in non-biliary pancreatitis [62]. A recent meta-analysis assessing the impact of obesity on AP in patients of Asian descent found a higher risk of severe pancreatitis, pancreatic necrosis, and organ failure in patients with a BMI of ≥30 kg/m² [62]. Imaging-based measures of visceral fat area (VFA) are even more predictive of health outcomes than BMI. CT image-based studies report that VFA and the VFA-to-skeletal muscle ratio correlate strongly with the severity of AP, systemic complications, intensive care unit (ICU) stay, and worse scores on prognostic indices, including Acute Physiology and Chronic Health Evaluation II (APACHE II), Bedside Index for Severity in Acute Pancreatitis (BISAP), Ranson, and SIRS scores [63,64]. Xie et al. reported adjusted hazard ratios exceeding 1.04 for a per-unit increase in VFA [63].

**Figure 2:**
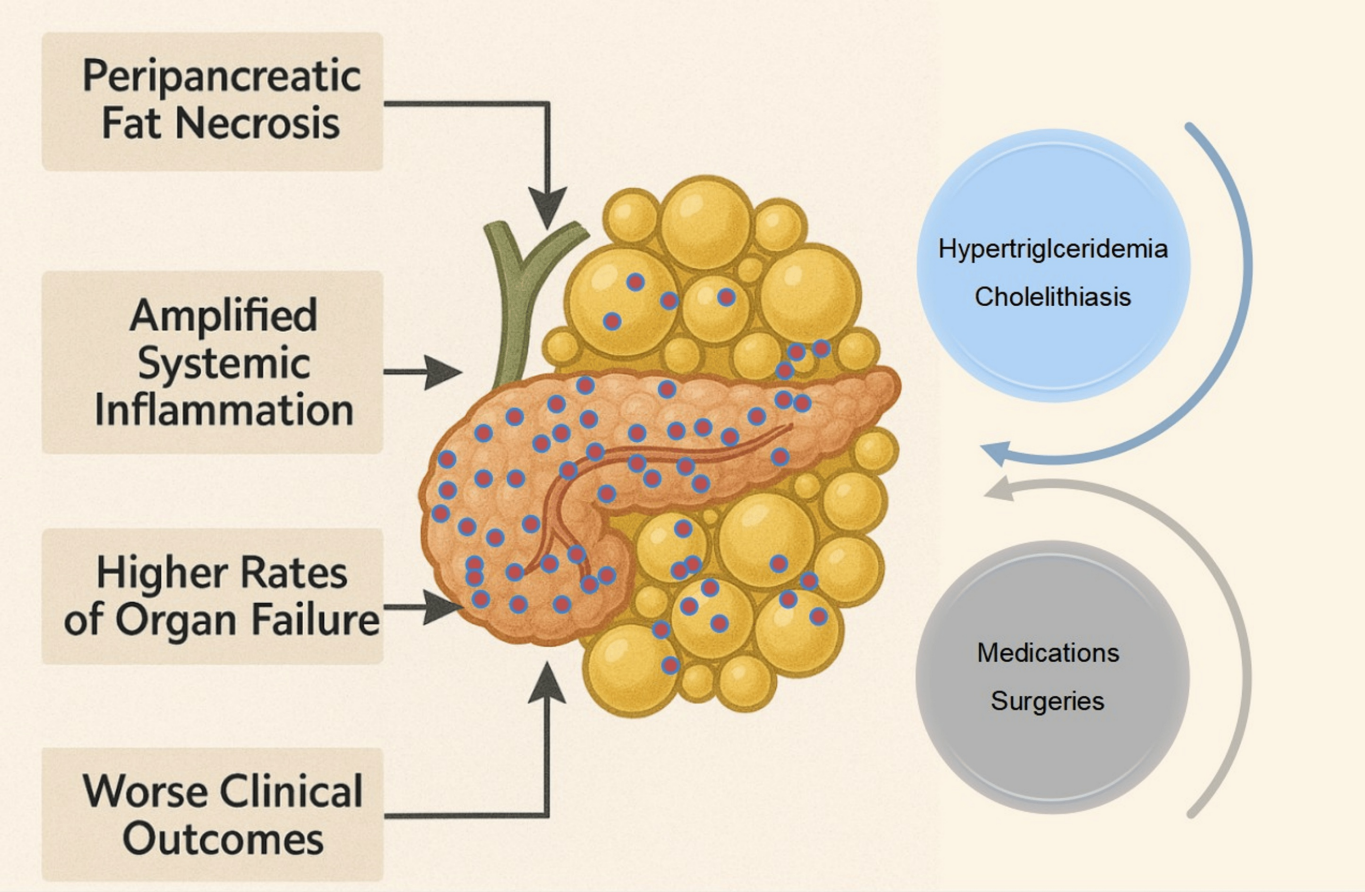
Acute pancreatitis and obesity. Obesity predisposes to acute pancreatitis through hypertriglyceridemia and increased prevalence of gallstone disease. Medical and surgical interventions for the treatment of obesity could also predispose to acute pancreatitis. The presence of obesity leads to increased fat necrosis and accentuated inflammatory response, culminating in a higher risk of organ failure and overall worse outcomes, in the setting of acute pancreatitis.

In conclusion, obesity is not only a risk factor for the development of AP but is also associated with increased risk of severe pancreatitis and complications (Figure 2). Based on the available literature, it is worthwhile to include obesity in prognostic models for AP. Future studies should focus on integrating obesity or related markers into predictive models for AP. Another area for potential research will be the role of adjunctive anti-lipase therapies in the setting of AP complicating obesity, as lipolysis and fatty acid release are key factors contributing to severity in this subset of patients.

Chronic pancreatitis

Chronic pancreatitis is characterized by progressive inflammatory injury and loss of pancreatic parenchyma, which is replaced by adipose and fibrous tissue. The progressive loss of parenchyma impairs exocrine and endocrine pancreatic function. In general, patients with chronic pancreatitis tend to have greater intrapancreatic fat, regardless of BMI, but this is considered a remodeling effect. The role of obesity in chronic pancreatitis, unlike in AP, is less clearly defined and even contradictory [65].

Obesity, particularly visceral adiposity, is closely linked to FP. Tirkes et al. demonstrated the correlation between visceral adiposity and pancreatic fat fraction in MRI. The study revealed an association between chronic pancreatitis and obesity, as well as pancreatic fat fraction [66]. Patients with FP were found to have a higher odds of developing chronic pancreatitis, with an adjusted odds ratio of 3.96 [67]. A recent Mendelian randomization study reported an association between genetically predicted intrapancreatic fat deposition and chronic pancreatitis, with an odds ratio of 1.64 [68]. The accumulation of intrapancreatic fat, associated with mechanical effects, lipotoxicity, oxidative stress, and chronic inflammation, may lead to pancreatic parenchymal injury. A long-term study involving patients with alcoholic chronic pancreatitis reported a high incidence of overweight before the onset of pancreatitis compared to the control population (54.2% versus 37.3%) [69].

Interestingly, underweight is also associated with poor outcomes in chronic pancreatitis. An evaluation of data from the Danish cohort reported higher mortality in patients with BMI <20 kg/m^2^, whereas BMI ≥25 kg/m^2^ was associated with better outcomes and reduced mortality [70]. However, this paradox can be explained primarily by the overall severity of the disease and poor nutritional status, which lead to undernutrition, a poor prognostic marker in most known human diseases. Obese patients tend to have higher metabolic reserve and less sarcopenia than their underweight counterparts, conferring the benefits [65]. Patients with chronic pancreatitis are prone to malnutrition and sarcopenia due to reduced oral intake, maldigestion, malabsorption, steatorrhea, frequent hospitalizations, and increased overall energy expenditure [71]. Studies have demonstrated lower muscle mass and functional status in patients with chronic pancreatitis, even with BMI in the overweight and obese range, compared to healthy counterparts. Hence, BMI alone is not considered an adequate method for assessing nutritional status in patients with chronic pancreatitis [72]. A meta-analysis of two studies demonstrated an inverse relationship between BMI and the risk of chronic pancreatitis: a 22% reduction in risk per five-unit increase in BMI [73].

The best-known etiological factors of chronic pancreatitis include alcohol and genetic factors. Numerous studies have recognized smoking as an independent risk factor in chronic pancreatitis. Studies have demonstrated a dose-dependent association between smoking and the development of chronic pancreatitis. Also, smoking is associated with progression of acute to chronic pancreatitis, chronic pancreatitis, and pancreatic adenocarcinoma [74]. Alcohol use and smoking are closely interlinked, with multiple studies and meta-analyses revealing the association irrespective of gender and ethnicity. This alcohol-smoking synergy has been demonstrated to increase the risk of pancreatitis [75]. It would be interesting to find out if obesity has similar synergistic effects on the pancreas in tobacco or alcohol users. Having said that, the impact of alcohol use and smoking on BMI continues to be debated, with studies showing positive and inverse correlation between them [76,77].

In summary, in the setting of chronic pancreatitis, weight reduction may be encouraged in obese and overweight individuals. Still, it should be tailored to avoid nutritional deficiencies, sarcopenia, and undernutrition as these could lead to poor overall outcomes and increased mortality.

Pancreatic neoplasia

The pancreas is the home to a variety of benign and malignant epithelial and neuroendocrine neoplasia. Pancreatic ductal adenocarcinoma, the most common among these, is infamous for late clinical presentation, aggressive tumor biology, limited treatment options, and high mortality. Globally, pancreatic cancer is the seventh leading cause of cancer-related death, with a five-year survival rate of less than 10% despite advancements in surgical and systemic therapies [78]. In the United States, pancreatic cancer is currently the third leading cause of cancer-related deaths, and it is expected to become the second leading cause of cancer-related deaths by the year 2030 [79,80].

Obesity has emerged as an independent and prevalent risk factor for pancreatic cancer, with epidemiological evidence linking increased BMI and central adiposity to both elevated cancer risk and poorer clinical outcomes [81]. It is estimated that, for every 5 kg/m² increase in BMI, the relative risk of developing pancreatic cancer increases by approximately 10-20%, while a 10 cm increase in waist circumference is associated with an 11% higher risk of pancreatic cancer [81]. Aune et al. also report an association of pancreatic cancer with duration of obesity [82]. Budek et al. also report an increased prevalence of pancreatic neuroendocrine tumors with obesity, though the impact is less clear and requires further elucidation [83]. Additionally, multiple case-control studies and cohort studies have reported a causal association between FP and pancreatic ductal adenocarcinoma [84].

Low-grade systemic inflammation, driven mainly by visceral adipose tissue, promotes cell proliferation, angiogenesis, and resistance to apoptosis, all of which favor malignant transformation [85]. Obesity promotes the expansion of myeloid-derived suppressor cells and regulatory T cells, while dampening the activity of cytotoxic T cells and natural killer (NK) cells, thereby further enabling tumor immune evasion and progression in pancreatic cancer [86,87]. Insulin resistance is associated with hyperinsulinemia and insulin-like growth factor-1 (IGF-1) levels, which activate downstream insulin receptor substrates and key oncogenic signaling cascades, including the PI3K/AKT/mTOR and MAPK/ERK pathways [88]. Preclinical studies by Zhang et al. have demonstrated the direct effect of insulin on pancreatic acinar cells via insulin receptors, promoting inflammation and early neoplastic changes [89]. Elevated insulin and IGF-1 levels correlate with higher pancreatic cancer risk and poorer prognosis [90].

A reduction in body weight and obesity has shown promise in attenuating the risk of pancreatic cancer. A meta-analysis, including over 3.7 million adults, reported a significantly lower risk of developing pancreatic cancer with metabolic-bariatric surgery regardless of diabetes status. However, the effects were more pronounced in patients with T2DM [91]. The GLP-1 receptor agonists have been associated with a significant reduction in pancreatic and other obesity-related cancers, likely due to their combined effects on weight loss, improved metabolic and inflammatory profiles, and direct anti-proliferative actions [92]. Also, obesity could potentially modulate the therapeutic response in patients with cancer [93].

Summarizing, obesity is a potentially modifiable risk factor in pancreatic cancerogenesis, and weight loss possibly confers benefit by attenuating the risk of pancreatic cancer.

Table 1 summarizes the implications of obesity on common pancreatic diseases.

**Table 1:** A summary of the detrimental effects of obesity on pancreas.

Impact of obesity on the pancreas and pancreatic diseases	
Fatty pancreas	Excess adipose tissue leads to lipid infiltration in pancreatic parenchyma, causing pancreatic steatosis and increased susceptibility to metabolic stress [8].
Endocrine dysfunction	Promotes β-cell dysfunction and insulin resistance, predisposing to impaired glucose tolerance, type 2 diabetes, and metabolic syndrome [8].
Acute pancreatitis	Obesity increases severity through peripancreatic fat necrosis, amplified systemic inflammation, higher rates of organ failure, and worse clinical outcomes [58,61,63].
Chronic pancreatitis	Accelerates progression due to persistent inflammation, fatty infiltration, and fibrosis; associated with higher complication rates [66,67].
Pancreatic cancer	Increases risk via chronic low-grade inflammation, insulin resistance, adipokine imbalance, and genetic/epigenetic changes promoting carcinogenesis [81,83].

## Conclusions

The effects of obesity on pancreatic health and diseases are profound and multifaceted. While the impact of obesity on acute pancreatitis and pancreatic cancer is well established, emerging data suggest that fatty pancreas and intrapancreatic adiposity may also contribute to chronic parenchymal injury. Lipotoxicity, oxidative stress, systemic inflammation, and metabolic dysregulation are proposed to play crucial roles in the pathogenesis of pancreatic disease in obesity. Weight reduction, lifestyle modification, pharmacological interventions, and bariatric surgery offer promising avenues to mitigate these risks; however, further robust clinical studies are warranted. Future research must prioritize the integration of obesity-related biomarkers into prognostic models and explore targeted therapies that address the obesity-pancreatic disease conundrum. Ultimately, addressing obesity not only improves overall health but also holds critical potential to alter the natural history of pancreatic disorders.
